# Volar locking plate versus external fixation for unstable distal radius fractures: a systematic review and meta-analysis based on randomized controlled trials

**DOI:** 10.1186/s12891-021-04312-7

**Published:** 2021-05-12

**Authors:** Qi Gou, Xiong Xiong, Dan Cao, Yuanliang He, Xu Li

**Affiliations:** 1 Department of Orthopedics, The First People’s Hospital of Longquanyi District, 610100 Chengdu, China; 2 Department of Anesthesiology, The First People’s Hospital of Longquanyi District, 610100 Chengdu, China

**Keywords:** Volar locking plates, External fixation, Putcome, Distal radius fractures, Meta-analysis, RCTs

## Abstract

**Background:**

The outcomes for volar locking plate (VLP) and external fixation (EF) in distal radius fracture cases remain controversial. The current study of randomized controlled trials (RCTs) aimed to assess VLP and EF, which might benefit distal radius fracture cases.

**Methods:**

RCTs comparing VLP and EF in distal radius fracture cases, until 18 March 2020, were systematically reviewed and summarized. The functional and radiographic outcomes, together with complications, for distal radius fracture cases, were evaluated.

**Results:**

In total, 12 studies comprising 1205 distal radius fracture cases were included. The VLP group had observed lower disability in the arm shoulder and hand score (DASH) at 3rd, 6th, and 12th -month post-operation, with the mean differences (MDs) of − 10.43 (95 % CI = − 15.77 to − 5.08, *P* < 0.01), − 3.48 (95 % CI = − 6.37 to − 0.59, *P =* 0.02), and − 4.13 (95 % CI = − 6.94 to − 1.33, *P* < 0.01), respectively. The VLP group also had lower visual analog scale scores (VAS) compared to the EF group, with MDs of − 0.10 (95 % CI = − 0.18 to − 0.03, *P* < 0.01) for the former at 6th -month post-operation. Also, the EF group exhibited better grip strength than that in the VLP group, with MD of 12.48 (95 % CI = 7.00–17.95, *P* < 0.01) at the 3rd month and 4.54 (95 % CI = 0.31–8.76, *P =* 0.04) at 6th month. No significant differences in radiographic outcomes were observed between the VLP and EF groups (*P* > 0.05). The VLP group had a lower complication rate than that in the EF group.

**Conclusions:**

VLP had a lower DASH score and VAS score but with lower grip strength. No significant differences in radiographic outcomes were observed. VLP had a lower complication rate than that of EF.

**Supplementary Information:**

The online version contains supplementary material available at 10.1186/s12891-021-04312-7.

## Background

Distal radius fracture is very common in the clinic and accounts for about 17 % of all types of fractures [1]. Bridging external fixation (EF), a technique aimed to obtain and maintain fracture alignment, has been widely used for many years. As a traditional and one of the most common treatment methods, EF can achieve acceptable results for distal radius fractures [2]. With about 50 % recurrent displacements and a 20–35 % complication rate [3, 4], in recent years, several new technologies have been developed for treating unstable distal radius fractures, one of which is named volar locking plates (VLP) [5].

Since the last decade, VLP are becoming more and more popular for surgical fixation of distal radius fractures [6]. The outcome for distal radius fractures includes malunion and highly unstable fractures [7], and the recent studies that assessed the use of fixed-angle screws within volar plates demonstrated that these can provide satisfactory stability by avoiding joint distraction [8, 9].

Several randomized studies were designed to assess the outcome of VLP versus EF for distal radius fracture cases [10, 11]. A meta-analysis [12] aimed to assess EF versus VLP in unstable fractures of distal radial concluded that cases treated with a VLP could obtain better functional outcomes. Another meta-analysis by Zhang et al. [13] reported a drastically opposing conclusion that the two methods had similar functional recovery. However, limited by the smaller sample sizes of the included studies, the two meta-analyses could not report more stratified analyses and could not conclude with more details. Very recently, several randomized controlled trials (RCTs) [14–17] addressing this topic provided new evidence, making it possible to update the results concerning this topic with powerful convincing. Therefore, we systematically conducted the current study to assess and compare the outcomes of VLP versus EF in unstable distal radius fractures.

## Methods

### Literature search

Widely used electronic datasets, such as PubMed, MEDLINE, Cochrane Library, and EMBASE databases, were used to search relevant articles until 18 March 2020. The individual and joint keywords of “distal radius”, “distal radial”, “fracture”, “fixator”, and “volar locking plate” were used to search potential articles according to the Preferred Reporting Items for Systematic Reviews and Meta-Analyses (PRISMA) statement [18]. With the aim of finding more relevant studies, studies and reviews on the same or similar topics were checked. Google Scholar was also employed to search potential studies.

### Eligibility criteria

The criteria to identify potential articles were: (1) RCTs that aimed to assess the outcome of VLP versus EF in unstable distal radius fractures, (2) the participants were cases clearly diagnosed with unstable distal radius fractures, (3) studies that reported functional outcome, (4) data of the characteristic outcome of participants could be extracted, (5) study was published in the English language, (6) the articles that reported more results were selected in the case when multiple populations were reported in an overlapping sample.

The studies were excluded if they were case reports, letters, brief reports, communications, reviews, non-randomized studies, non-human studies, and studies that were published in a language other than English.

Moreover, unstable distal radius fractures were defined either as [12] the fracture fragments being redisplaced following closed reduction and cast immobilization, or those fulfilling any three of the following criteria: dorsal angulation more than 20°, dorsal comminution, an intra-articular fracture, an associated ulnar styloid fracture, and age more than 60 years. Major complications were those leading to a reoperation, permanent nerve injury, or a persistently reduced level of function, while the minor complications were defined as either transient or those that did not affect one’s final level of function.

### Data obtaining

All the studies obtained initially from the electronic databases were evaluated by two investigators independently. All necessary data and information were extracted using a standardized form independently by the same two investigators. All discrepancies on the data that could not be solved by consensus were discussed and settled with another reviewer. For each included study, the following data were extracted: article characteristics (e.g., the first author, publish year, and study design), participant features (age, sex, and sample size,), therapy characteristics of VLP and EF, and outcome characteristics.

### Bias risk assessment

The bias risk assessment tool, Cochrane Collaboration’s Risk of Bias tool [19], was employed for each study. The bias risk of each study was graded into three categories following the guideline from lower to higher. The detailed assessment process included the following seven items: (1) sequence generation, (2) allocation concealment, (3) blinding for participants, (4) blinding for outcome assessment, (5) assessment of incomplete results, (6) completeness on presenting the data, and (7) other biases. The item for, which insufficient information was provided for them to be award a low or high risk of bias, the relevant items were judged as unclear.

### Assessment of the quality of studies

 The two reviewers (*names of two authors)* stated above independently evaluated the quality of evidence following the Jadad scale [20]. Each included study was scored from 0 to 5 according to their performance on the three items of the Jadad scale – randomized on selecting participants, blinded for grouping, and accountable for every participant. For the “randomization” and “blinding” item, one or two stars would be given for the “yes” response, and one star would be given for the answer “yes” for “accountability”. For setting a minimum standard for the inclusion of a study in the current study, the study with one or two stars was deemed as having low quality [21].

### Statistical analysis

Discrete variables, such as complications of each group, were estimated and pooled by risk ratio (RR) and relevant 95 % confidence interval (CI). Mean difference (MD) and 95 % CI were employed to pool continuous variables, such as wrist range of motion (WRM), etc. All the pooled variables were subjected to an inverse variance procedure with a random model. The *I*^*2*^ statistic was used to assess the heterogeneity in each analysis. Heterogeneity in each analysis process was identified as lower (*I*^*2*^ less than 25 %), moderate (*I*^*2*^ between 25 and 50 %), and higher (*I*^*2*^ more than 50 %) [22]. For the process with 50 % ≤ *I*^*2*^, the studies were seriatim excluded from the analysis. Begg’s rank correlation [23] and Egger’s weighted regression method [24] were employed to evaluate the publication biases in the analysis processes. Stratified analyses were conducted based on the characteristics of the participants and the outcomes of each group. Pooled processes and forest plots were completed using Review-Manager (version 5.2, The Cochrane Collaboration, Oxford, UK). The publication bias was evaluated with STATA 15.1 (Stata Corporation, College Station, TX, USA). For all analyses, the *P*-value of less than 0.05 was deemed as statistically significant.

## Results

### Study inclusion

**Fig. 1:**
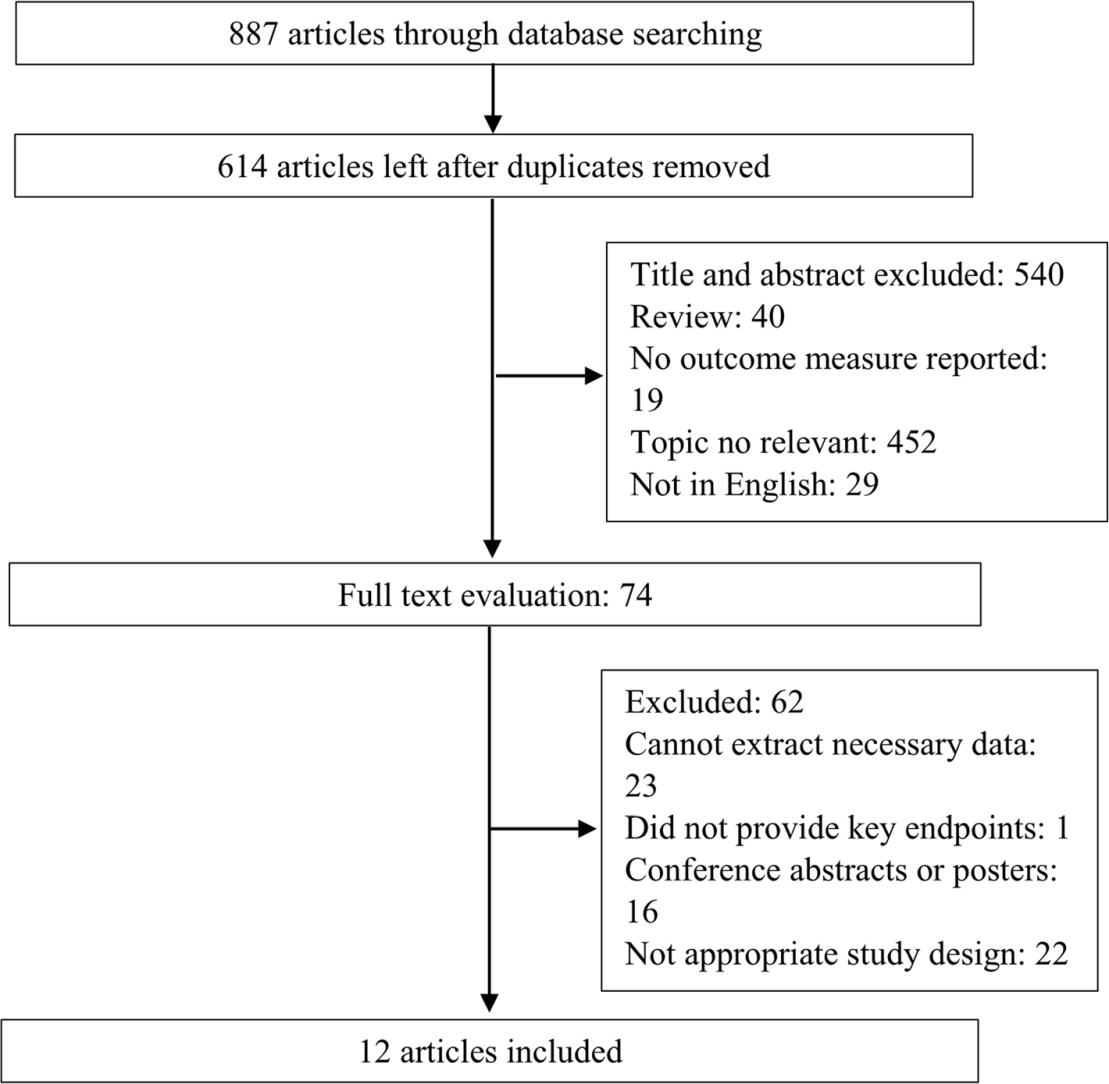
Flow chart of the study selection process

Ultimately, 887 articles were included after the initial search in the electronic datasets, among which 273 were removed due to duplication. Most of the remaining articles were removed by reading the titles or abstracts. Finally, 12 studies [10, 11, 14–17, 25–30] were included in the current study by browsing 74 full-text manuscripts. The flow chart for the literature selection process can be found in Fig. 1.

### Study characteristics

A total of 1205 distal radius fracture cases were included in the current study. The 12 studies were conducted in the United States [25, 26, 30] (*N* = three), Sweden [10, 16] (*N* = two), Norway [17, 28] (*N* = two), France [17] (*N* = one), Germany [27] (*N* = one), United Kingdom [29] (*N* = one), India [14] (*N* = one), and the Republic of Korea [15] (*N* = one). The sample sizes of the 12 studies ranged between 34 and 134. The features of the participants are presented in Table 1.

**Table 1:** Characteristics of the included studies

Study included	Country	Age, years (range, VLP/EF)	Male (%) (VLP/EF)	N (VLP/EF)	Follow-up period (month)	AO classification (% of A/B/C)	Intervention	
							**VLP**	**EF**
Egol, et al., 2008 [25]	United States	52.2 (19–87)/ 49.9 (18–78)	61/62	39/38	12	37/4/59	VLP	Bridging EF ± K-wires
Wei, et al., 2009 [26]	United States	61.0 ±18.0/ 55.0 ±16.0^a^	25/28	12/22	12	45/0/55	Locked radial column plates and VLP	Bridging EF ± K-wires
Wilcke, et al., 2011 [10]	Sweden	55.0 (20–69)/ 56.0 (21–69)	24/23	33/30	12	76/0/24	VLP	Bridging EF ± K-wires
Jeudy, et al., 2012 [11]	France	64.7 ±3.7/ 64.6 ±3.5^a^	28/21	36/39	24 weeks	0/0/100	Volar fixed angle plates	Bridging EF ± K-wires
Gradl, et al., 2013 [27]	Germany	63 (18–88)	13 ^b^	52/50	12	61/0/39	Volar fixed angle plates	Non-bridging EF ± K-wires
Karantana, et al., 2013 [29]	United Kingdom	48.0 ±15.0/ 51.0 ±16.0^a^	39/22	66/64	12	42/52/6	VLP	Bridging EF ± K-wires
Williksen, et al., 2013 [28]	Norway	54.0 (20–84)	20 ^b^	52/59	52 weeks	26/0/74	Volar fixed angle plates	Bridging EF ± K-wires
Shukla, et al., 2014 [14]	India	39.3 ±13.1/ 39.0 ±13.1^a^	42/47	36/38	12	NA	VLP	Bridging EF
Roh, et al., 2015 [15]	South Korea	54.4 ±10.9 /55.3 ±11.2^a^	70/64	48/62	12	0/0/100	VLP	Bridging EF ± K-wires
Navarro, et al., 2016 [16]	Sweden	63.0 (51.0–74.0)/ 63.0 (50.0–74.0)	37/34	69/65	12	40/0/60	VLP	Bridging EF ± K-wires
Chung, et al., 2019 [30]	United States	67.3 ±6.2/ 69.5 ±8.4^a^	15.4/7.8	65/64	12	NA	VLP	Bridging EF
Hammer, et al., 2019 [17]	Norway	56.0 ±10.5/ 54.0 ±12.4^a^	30/33	84/82	12	0/0/100	VLP	Bridging EF ± K-wires

Abbreviations: *EF* external fixation, *VLP* volar locking plates, *NA* not available

^a^means ± standard deviation

^b^overall results

### Risk of bias and quality assessment

**Fig. 2:**
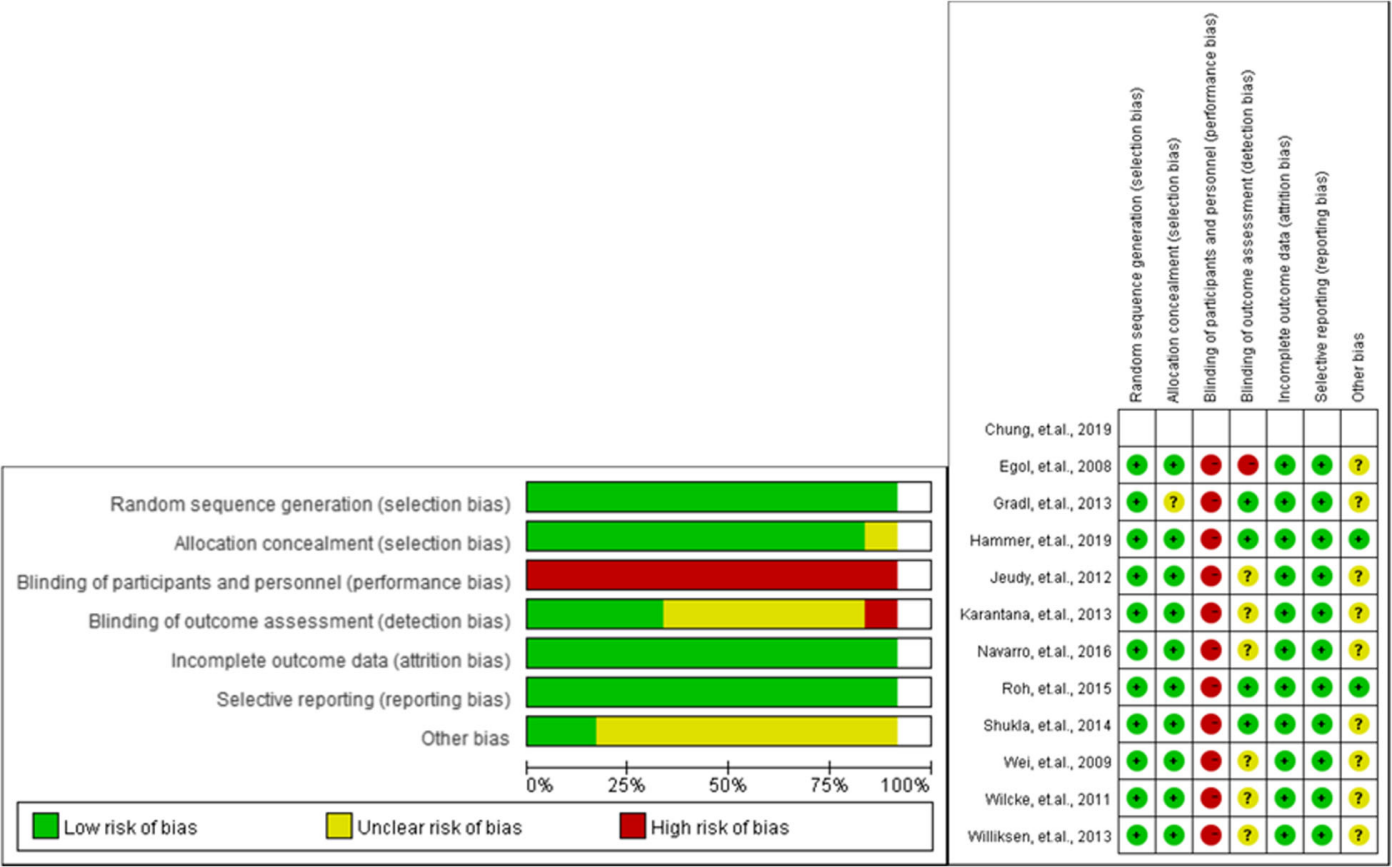
Risk of bias assessment of the included studies

The majority of the included studies were evaluated as those with an acceptable risk of bias and quality. The majority of the studies were assessed as having a score of 3 on the Jadad scale as some of them did not conduct blinding methods. More detailed results on the risk of bias and quality assessment can be found in Fig. 2 and Supplementary Table 1, respectively.

### DASH scores (disabilities of the arm shoulder and hand score)

**Fig. 3:**
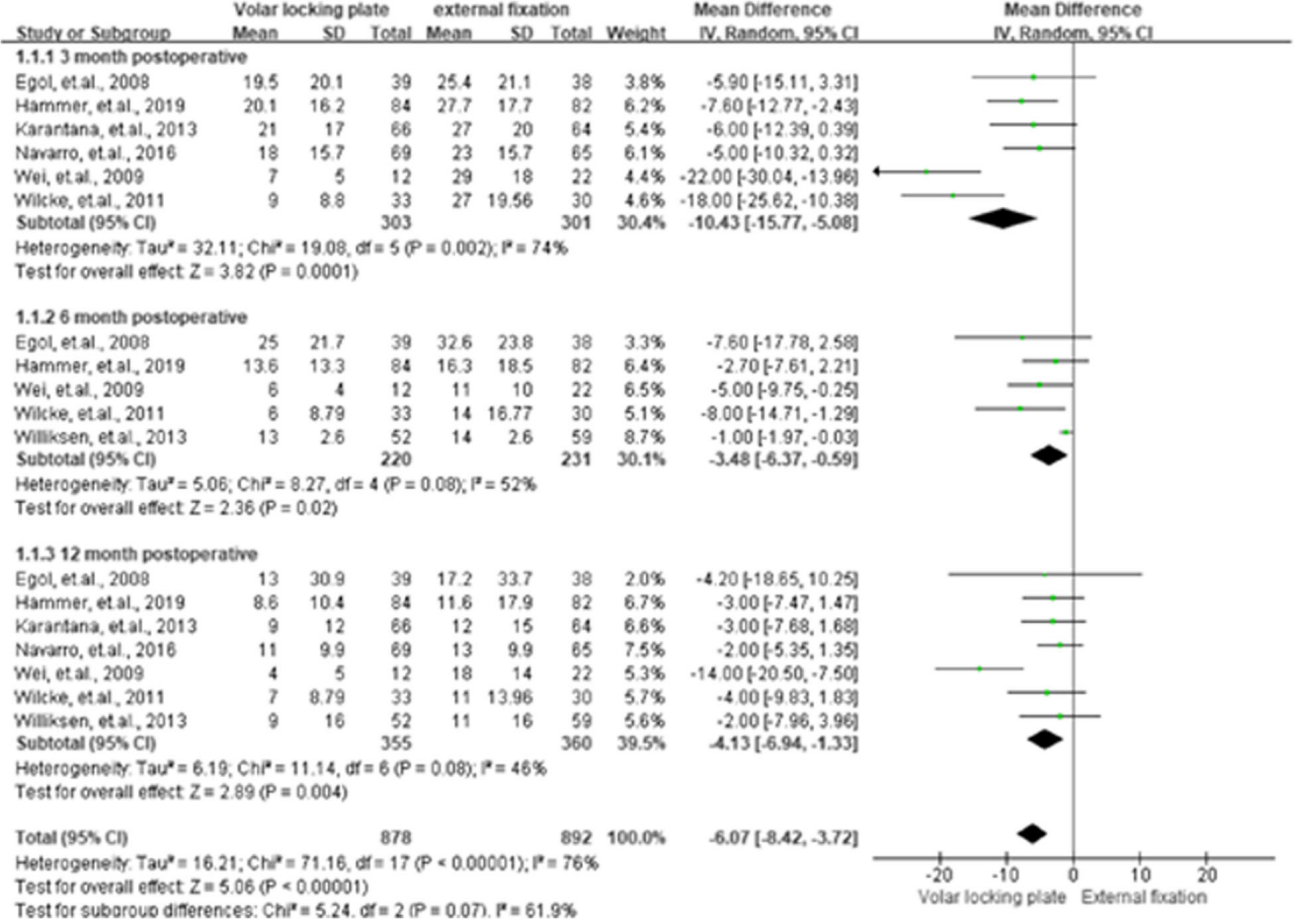
Summarized disabilities of the arm shoulder and hand score

Seven RCTs [10, 16, 17, 25, 26, 28, 29] with 715 cases reported data on DASH scores. The DASH scores for VLP were statistically lower when compared with EF at 3rd, 6th, and 12th month postoperation, with MDs of − 10.43 (95 % CI = − 15.77 to − 5.08, *P* < 0.01), − 3.48 (95 % CI = − 6.37 to − 0.59, *P =* 0.02), and − 4.13 (95 % CI = − 6.94 to − 1.33, *P* < 0.01), respectively. The summarized results were assessed as having a slightly higher or moderate heterogeneity, with *I*^2 =^ 74 %, 52 %, and 46 % for the 3rd, 6th, and 12th -month post-operation. The summarized results of the DASH scores are presented in Fig. 3.

### Visual analog scale scores

**Fig. 4:**
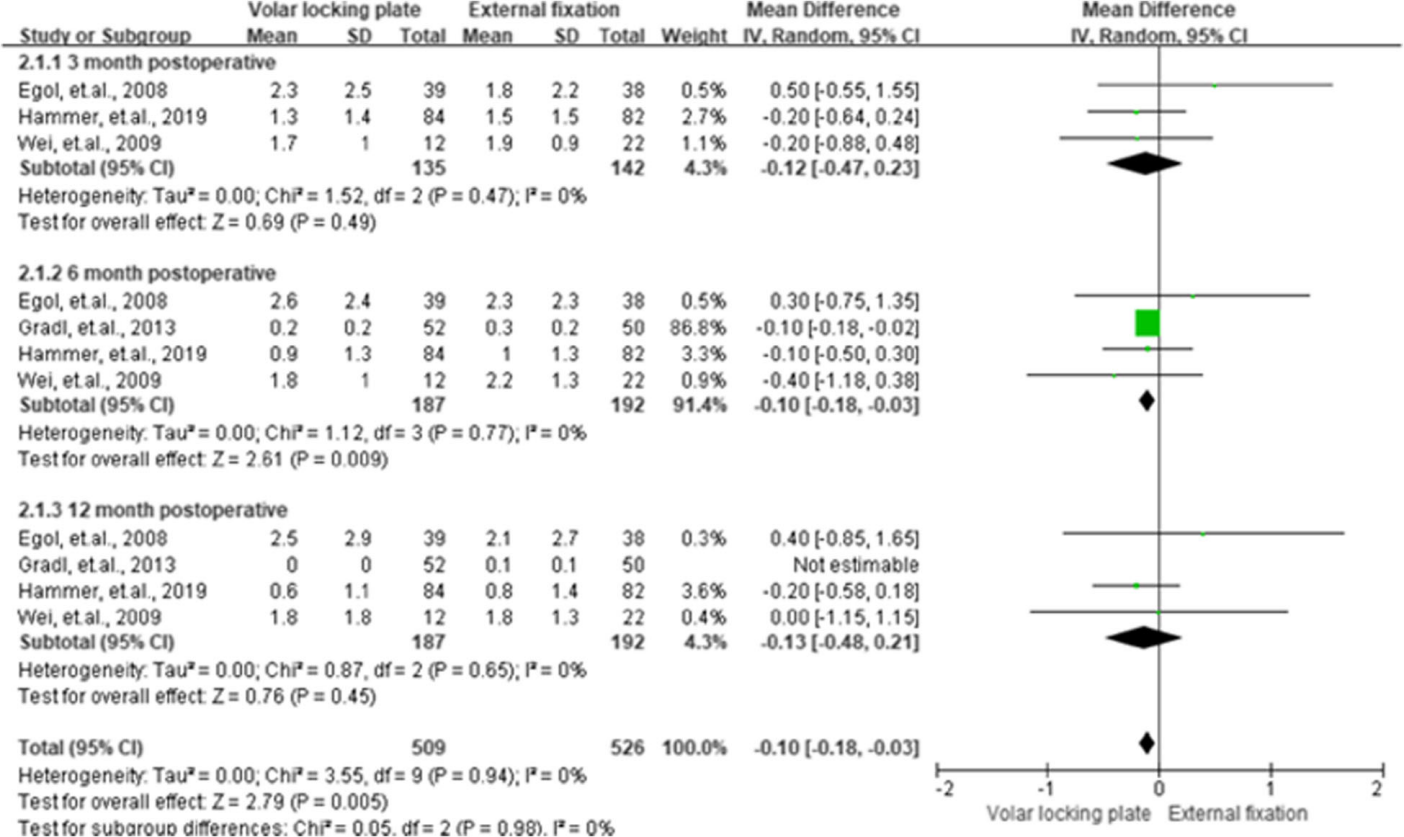
Summarized visual analog scale scores

As shown in Fig. 4, of the twelve RCTs, four studies [17, 25–27] provided data on VAS (visual analog scale scores) and included 379 cases. No heterogeneity (퐼^2^ = 0 %) for the scores of 3rd, 6th, and 12th -month post-operation was found. The VLP group had a better VAS than the EF group, with MD of − 0.10 (95 % CI = − 0.18 to − 0.03, *P* < 0.01) for the former at 6th -month post-operation.

### Grip strength (GS) for the uninjured side

**Fig. 5:**
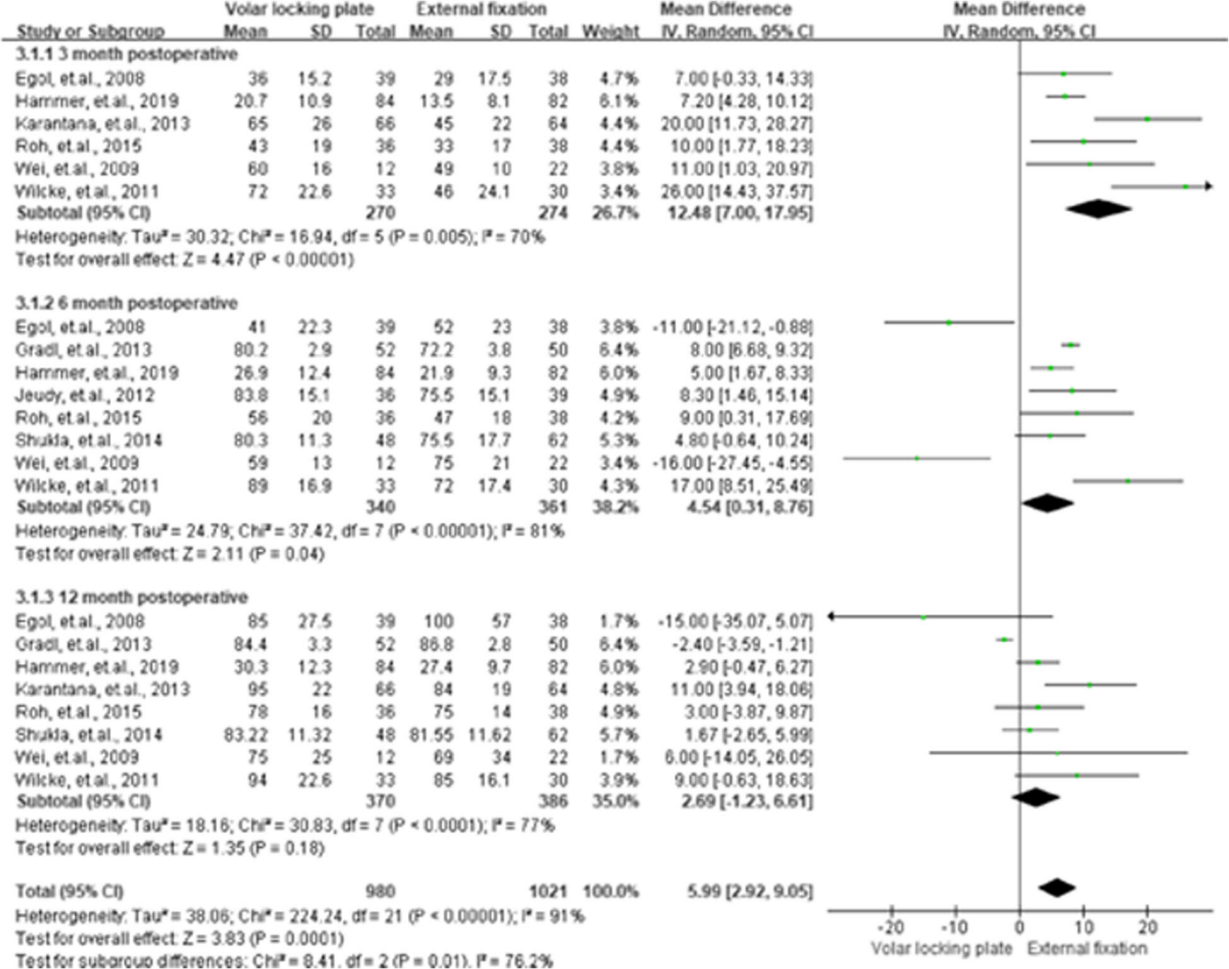
Summarized grip strength results

As shown in Fig. 5, eight studies [10, 14, 15, 17, 25–27, 29] provided and assessed the detailed GS of the VLP and EF groups for the 3rd, 6th, and 12th -month post-operation. For the pooled MD of the two groups, all the summarized results were assessed as having higher heterogeneity (*I*^2 =^ 70 %, 81 %, and 77 %), and the VLP group demonstrated a significantly lower GS than that of the EF group, with MD of 12.48 (95 % CI = 7.00–17.95, *P* < 0.01) at the 3rd month and 4.54 (95 % CI = 0.31–8.76, *P =* 0.04) at the 6th month for the former.

### WRM

**Fig. 6:**
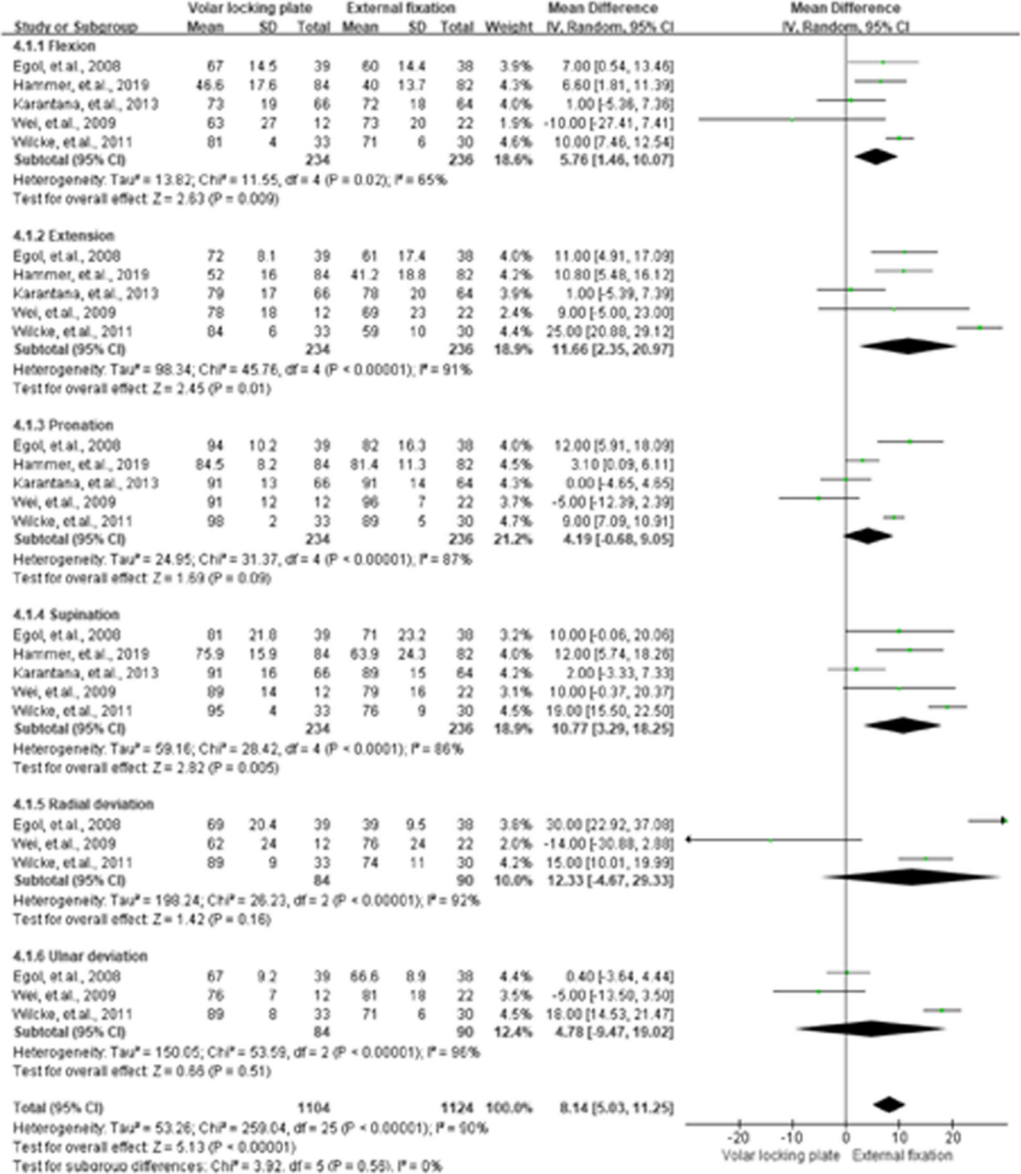
Summarized wrist range of motion results after 3 months

**Fig. 7:**
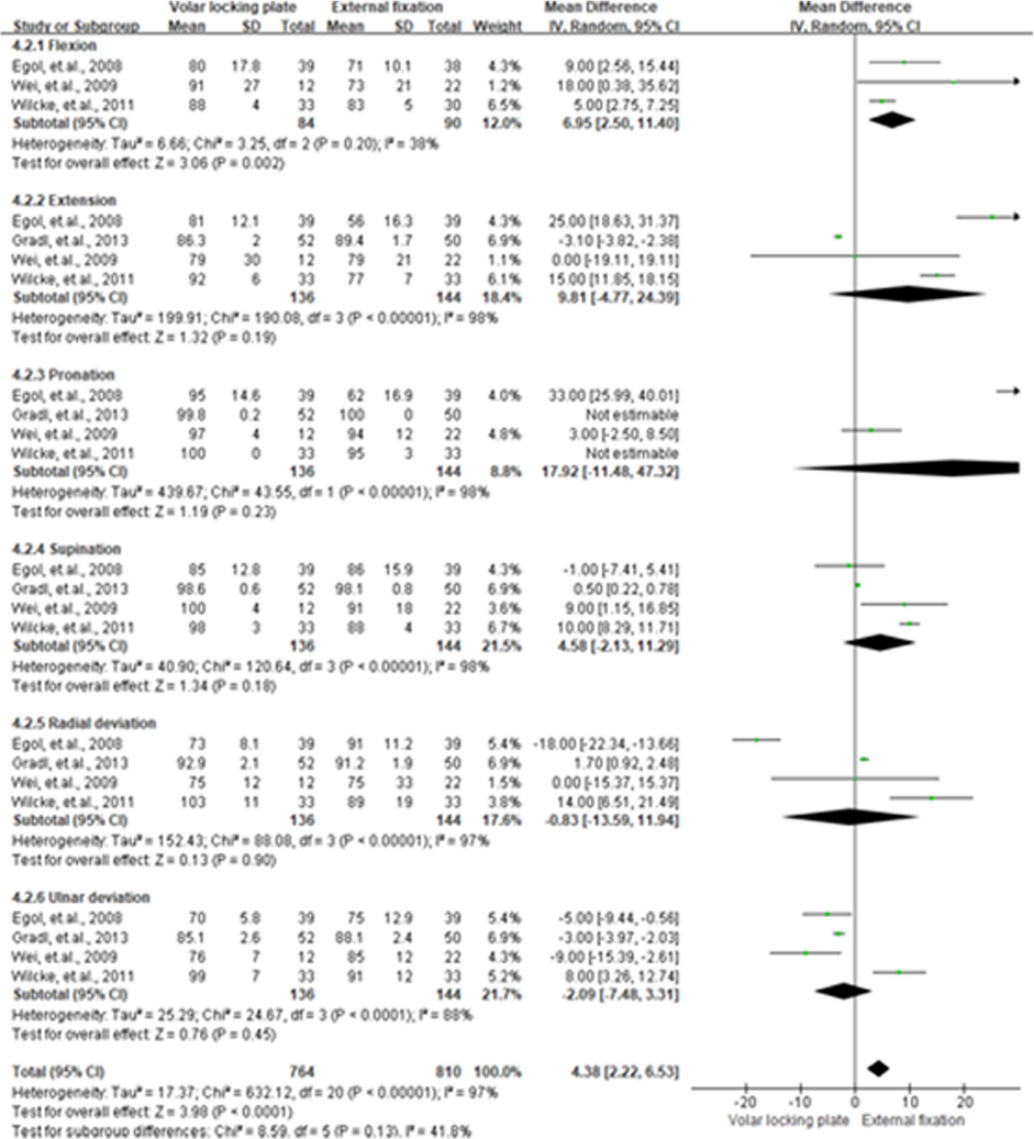
Summarized wrist range of motion results after 6 months

**Fig. 8:**
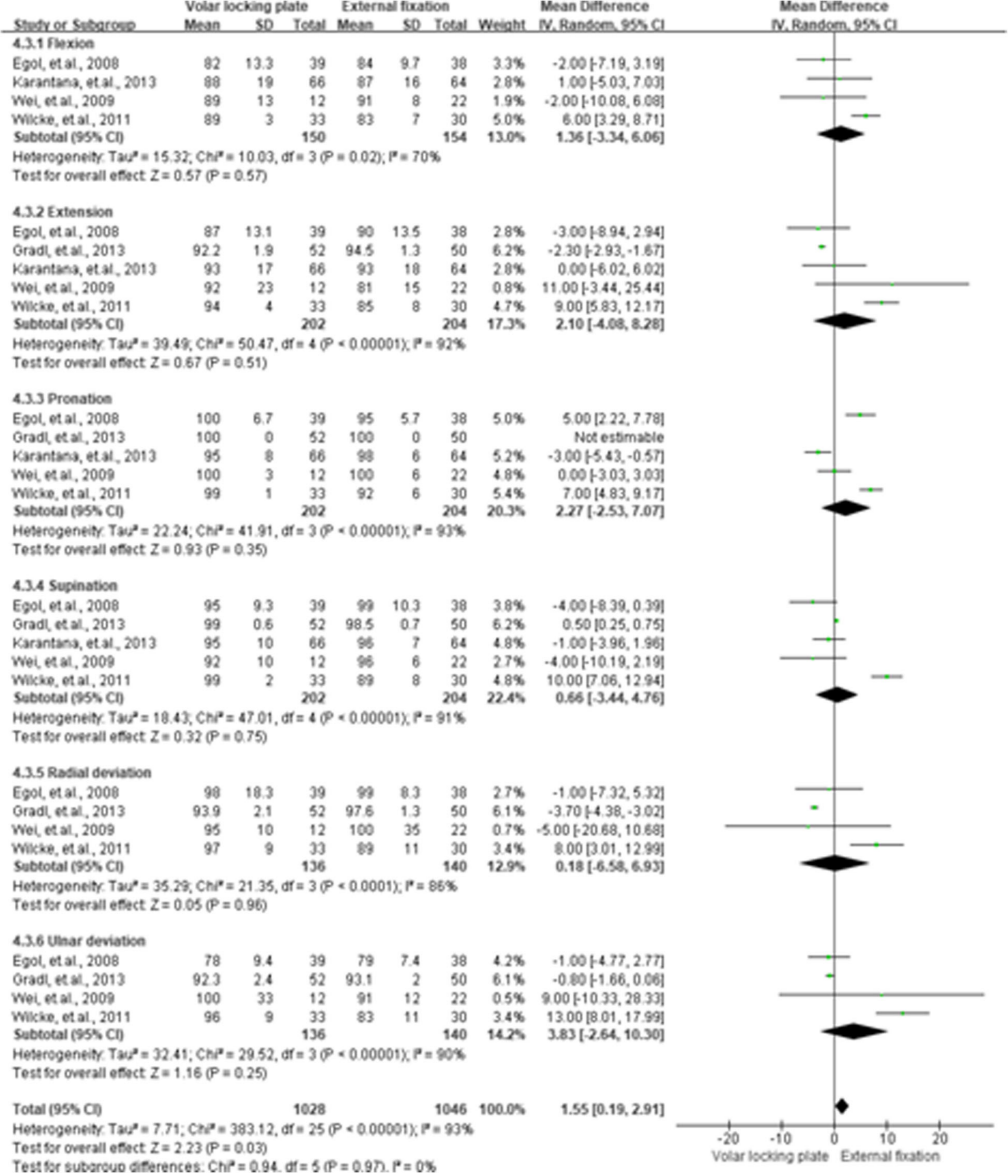
Summarized wrist range of motion results after 12 months

The WRM was presented in six categories of pooled flexion, extension, pronation, supination, radial deviation, and ulnar deviation. For the 3rd month, flexion (MD = 5.76, 95 % CI = 1.46–10.07, *P* < 0.01), extension (MD = 11.66, 95 % CI = 2.35–20.97, *P =* 0.01), and supination (MD = 10.77, 95 % CI = 3.29–18.25, *P* < 0.01) had better performance in the VLP group than that in the EF group. Results of WRM for the 3rd, 6th, and 12th month are shown in Figs. 6, 7 and 8, respectively.

### Radiographic outcomes

**Fig. 9:**
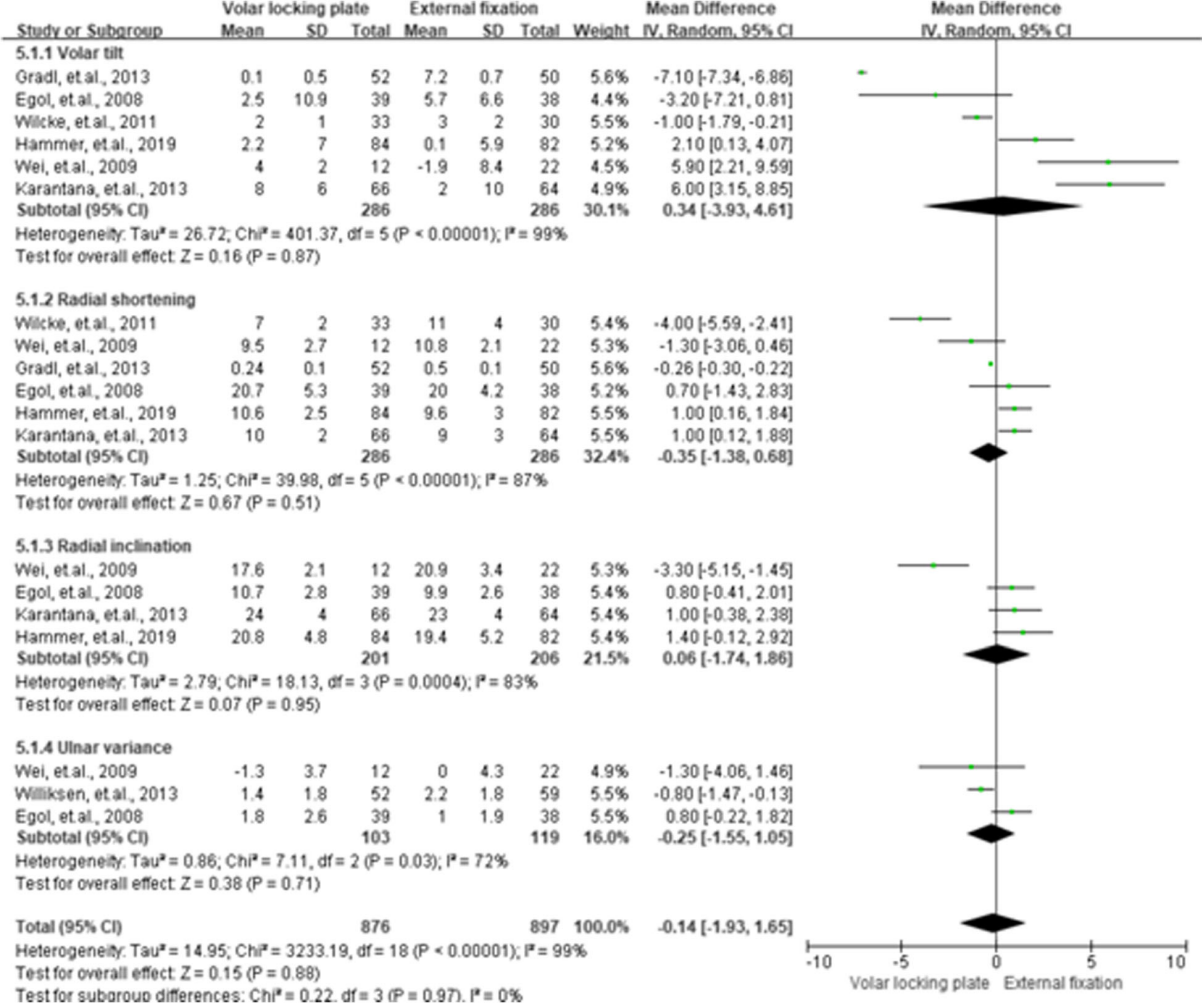
Summarized radiological measurement results

The outcomes on radiographic variables were evaluated at the 12th month and are presented in Fig. 9. No significant differences were observed between VLP and EF (*P* > 0.05) for the results on volar tilt, radial shortening, radial inclination, and ulnar variance.

### Complications

**Fig. 10:**
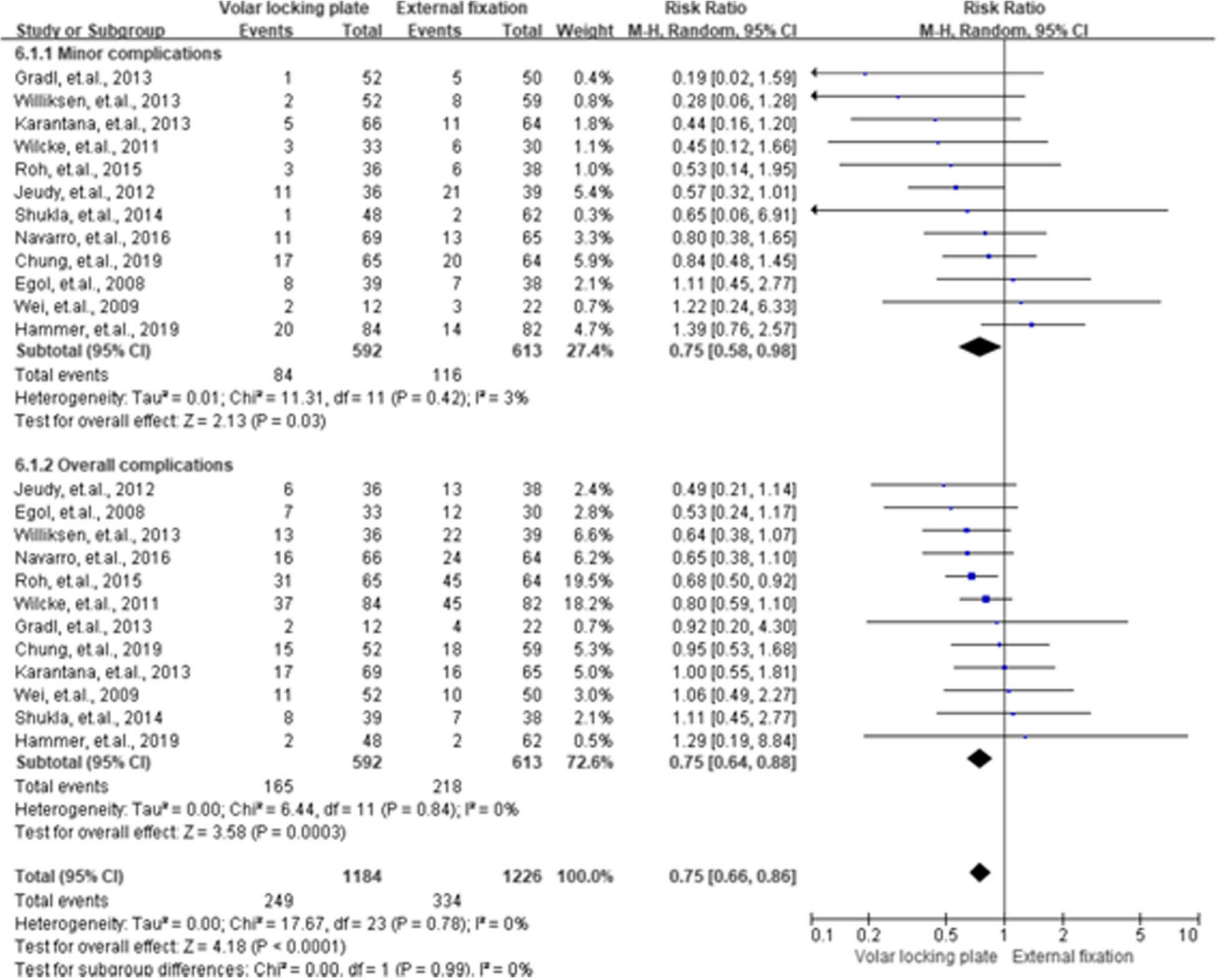
Summarized complications

All the 12 included RCTs reported the complication rate, and the results can be found in Fig. 10. The pooled result on complication revealed that VLP led to a lower proportion of complication compared to EF, with RR of 0.75 (95 % CI = 0.58–0.98, *P* = 0.03) for the former. For the summarized minor complication rate, the results were similar (RR = 0.75, 95 % CI = 0.64–0.88, *P* < 0.01).

### Heterogeneity analyses

Sensitivity analyses were performed by excluding studies seriatim to explore the sources of heterogeneity. In the pooled analysis of DASH, GS, WRM, and radiographic outcomes, the heterogeneity exhibited became significantly lower by excluding one or two studies. The detailed results are presented in Supplementary Figs. 1, 2, 3, 4, 5 and 6.

### Publication bias

No publication bias was found with Begg’s rank correlation and Egger’s weighted regression analysis (all *P* < 0.05). The p-values for all pooled analyses are presented in Supplementary Table 2.

## Discussion

In our current study on VLP versus EF for distal radius fracture cases, 12 RCTs with 1205 cases were included. Compared with EF, VLP might involve a lower DASH score and VAS score. In regard to WRM, VLP was better than EF in flexion and supination. However, EF significantly increased GS compared to VLP. No differences were observed for the radiographic parameters. For the pooled minor complication rate and total complication rate, VLP presented a slightly lower proportion of complication compared to EF.

Another study [31] addressing a similar topic observed that VLP could obtain better subjective scores and radiographic parameters. By including more RCTs with more participants, the conclusions for subjective scores would be comparable. However, the DASH scores for VLP at three, six, and twelve months exhibited a downward trend. One of the significant differences in the outcomes of VLP and EF was the DASH score in 3rd month. The result suggested that cases treated with VLP might obtain a better functional outcome at earlier stages. However, the difference in GS revealing a better outcome for EF at three or six months suggested an advantageous recovery for GS in the early rehabilitation period, while the long-term outcomes were similar. This may be attributed to the earlier rehabilitation and exercise in the group [25].

However, no differences were observed for the radiographic outcomes. With a significantly larger number of included studies conducted in various countries, the results were different from those of the meta-analysis reported by Gouk et al. [31] and Zhang et al. [32] and were similar to those of a prospective randomized trial [25].

Common complications after EF were transient superficial nerve palsy, pin track infection, and loss of fracture reduction [25, 26], while for VLP, they were median nerve palsy, flexor tenosynovitis, extensor tenosynovitis, and tendon rupture [25, 26]. In our study, a significant difference was observed when assessing the overall and minor complications. In another systematic review aimed to assess the complications of distal radial fractures in the elderly population, small but clinically unimportant differences in the clinical outcomes were reported between EF and VLP [33], in addition to a higher reoperation rate after plate fixation. Moreover, VLP was reported to be associated with more complications requiring late secondary surgery, which occurred 2 to 7 years after the fracture surgery [34, 35]. Therefore, it is important to consider the burden of complications, especially for those beyond one year after the distal radius fracture surgery. For the clinical settings, small clinical advantages might not necessarily compensate for higher costs or higher risks of complications. Although providing a stable fixation, VLP may compress and damage the flexor tendons [36]. Aiming to avoid flexor tendon problems, in clinical settings, surgeons always recommend keeping the plates proximal to the volar rim. Moreover, some highly comminuted and distal fractures may not be amenable to VLP, such as fractures with multiple intra-articular fragments in the distal bone [37]. The watershed line needs to be considered for VLP. It could be an indication point for the plate placement that delimits the concave structure of the radius distal volar face [37, 38]. VLP applied distally to that point may cause tendon complications [37, 38]. Therefore, the current meta-analysis might provide references for clinicians when dealing with distal radius fractures. Although with various AO fracture types, the results remain similar. The result was consistent with the study conducted by Hoffmann et al., who compared the AO type B and C fractures of the distal radius. Patients with Type-C volar might experience more pain during early recovery, but ultimately their outcome was comparable with that of patients with a Type-B.

However, certain limitations of the current study should be considered when drawing conclusions. First, the majority of the included studies had limited participants and most of the studies were conducted in western countries. Therefore, we could not conduct more subgroup or sensitivity analyses. Second, when including the participants in the two groups, the majority of the researchers did not match the cases for the two groups by age or gender. The differences in age and the gender ratio in the case and control groups might cause heterogeneities and reduce reliability. Third, the process of evaluating the outcomes and the definition of the outcomes were relatively promiscuous. More than ten scores or methods were used to assess or define the outcomes. The follow-up time for assessing the outcomes in the studies ranged from 2 weeks to 5 years. Therefore, we could not have more pooled results on functional outcomes with limited studies. Fourth, none of the included studies reported comorbidities in the participants. The comorbidities might also lead to or contribute to adverse outcomes. Therefore, the various potential comorbidities might also cause heterogeneities and even reduce the reliability of the results.

## Conclusions

The current study assessed VLP versus EF in terms of the outcomes for distal radius fractures. VLP was observed to have a better DASH score, VAS score, and part of WRM, but a lower GS. Regarding the radiographic outcomes, VLP and EF demonstrated similar results. However, cases might have a lower complication rate when treating with VLP. VLP might benefit the cases more than EF and could be a preferential surgical technique for distal radius fracture patients. In the future, RCTs with a larger sample size and RCTs with matched characteristics of the cases and severity degrees of the patients are needed to detect more potentially important differences.

## Supplementary Information

**Additional file 1.**

**Additional file 2: Supplementary Figure1.** Heterogeneityanalysis for summarized disabilities of the arm shoulder and hand score. **Supplementary Figure2. **Heterogeneityanalysis for summarized grip strength. **Supplementary Figure3. **Heterogeneityanalysis for summarized wrist range of motion after 3 months. **Supplementary Figure4. **Heterogeneityanalysis for summarized wrist range of motion after 6 months. **Supplementary Figure5. **Heterogeneityanalysis for summarized wrist range of motion after 12 months. **Supplementary Figure6. **Heterogeneityanalysis for summarized radiological measurement. **Supplementary Table 1. **Quality assessment ofincluded studies by Jadad score. **Supplementary Table 2. **Publication bias ofsummarized outcomes.

## Data Availability

All data generated or analyzed during this study are included in this article [and its supplementary information files].

## Abbreviations

VLP: Volar locking plate
EF: External fixation
RCT: Randomized controlled trials
RR: Risk ratio
CI: Confidence interval
MD: Mean difference
WRM: Wrist range of motion
GS: Grip strength
