# Supplementary material for: Volar locking plate versus external fixation for unstable distal radius fractures: a systematic review and meta-analysis based on randomized controlled trials
Source: BMC Musculoskelet Disord. 2021 May 12;22:433. doi: 10.1186/s12891-021-04312-7 (PMC8117612; doi:10.1186/s12891-021-04312-7)
Supplement: Supplementary file 1 — Additional file 1. [file 12891_2021_4312_MOESM1_ESM.docx]

**PubMed: 254 results (inception to 18 March 2020)**

1. ((((distal[Title/Abstract]) AND fracture*[Title/Abstract]) AND ((radius[Title/Abstract]) OR radial[Title/Abstract])) OR (((((colles’ fracture*[Title/Abstract]) OR colles fracture*[Title/Abstract]) OR smith fracture*[Title/Abstract]) OR barton fracture*[Title/Abstract]) OR wrist fracture*[Title/Abstract]))
2. (((volar[Title/Abstract]) OR palmar[Title/Abstract]) OR palmer[Title/Abstract]) AND ((((external fix*[Title/Abstract]) OR fixation ext*[Title/Abstract]) OR fixateur ext*[Title/Abstract]) OR fixator ext*[Title/Abstract])
3. 1 AND 2

**EMBASE: 341 results (inception to 18 March 2020)**

1. ((((distal.ti,ab) AND fracture*.ti,ab) AND ((radius.ti,ab) OR radial.ti,ab)) OR (((((colles’ fracture*.ti,ab) OR colles fracture*.ti,ab) OR smith fracture*.ti,ab) OR barton fracture*.ti,ab) OR wrist fracture*.ti,ab))
2. (((volar.ti,ab) OR palmar.ti,ab) OR palmer.ti,ab) AND ((((external fix*.ti,ab) OR fixation ext*.ti,ab) OR fixateur ext*.ti,ab) OR fixator ext*.ti,ab)
3. 1 AND 2

**Cochrane Library: 341 results (inception to 18 March 2020)**

1. (distal:ti,ab,kw and fracture*:ti,ab,kw) AND (radius:ti,ab,kw or radial:ti,ab,kw or “Colles’ fracture*”:ti,ab,kw or “Colles fracture*”:ti,ab,kw or “Barton’s fracture”:ti,ab,kw or smith fracture*:ti,ab,kw or “Smith’s fracture*”:ti,ab,kw or wrist fracture*:ti,ab,kw)
2. (“volar”:ti,ab,kw or “palmar”:ti,ab,kw or “Palmer”:ti,ab,kw) AND (extern*:ti,ab,kw or “fixation”:ti,ab,kw or “fixator”:ti,ab,kw or fixat*:ti,ab,kw)
3. 1 AND 2
