# Supplementary material for: Volar locking plate versus external fixation for unstable distal radius fractures: a systematic review and meta-analysis based on randomized controlled trials
Source: BMC Musculoskelet Disord. 2021 May 12;22:433. doi: 10.1186/s12891-021-04312-7 (PMC8117612; doi:10.1186/s12891-021-04312-7)
Supplement: Supplementary file 2 — Additional file 2: Supplementary Figure1. Heterogeneityanalysis for summarized disabilities of the arm shoulder and hand score. Supplementary Figure2. Heterogeneityanalysis for summarized grip strength. Supplementary Figure3. Heterogeneityanalysis for summarized wrist range of motion after 3 months. Supplementary Figure4. Heterogeneityanalysis for summarized wrist range of motion after 6 months. Supplementary Figure5. Heterogeneityanalysis for summarized wrist range of motion after 12 months. Supplementary Figure6. Heterogeneityanalysis for summarized radiological measurement. Supplementary Table 1. Quality assessment ofincluded studies by Jadad score. Supplementary Table 2. Publication bias ofsummarized outcomes. [file 12891_2021_4312_MOESM2_ESM.docx]

***Supplementary information***

**Volar locking pla****te versus external fixation for distal radius fractures: a systematic review and meta-analysis based on randomized controlled trials**

**Running title:** VLP versus EF

Qi Gou^1,#^, Xiong Xiong^1,#^, Dan Cao^2^, Yuanliang He^1,*^, Xu Li^1,*^

^1^Department of orthopedics, The first people's hospital of longquanyi district, Chengdu, 610100, China

^2^Department of anesthesiology, The first people's hospital of longquanyi district, Chengdu, 610100, China

^#^ Qi Gou and Xiong Xiong contributed equally to this paper.

***Correspondence:**

Yuanliang He, Department of orthopedics, The first people's hospital of longquanyi district, Chengdu, 610100, China

Email: yuanlianghe88@126.com

Xu Li, Department of orthopedics, The first people's hospital of longquanyi district, Chengdu, 610100, China

Email: lixu1988med@126.com


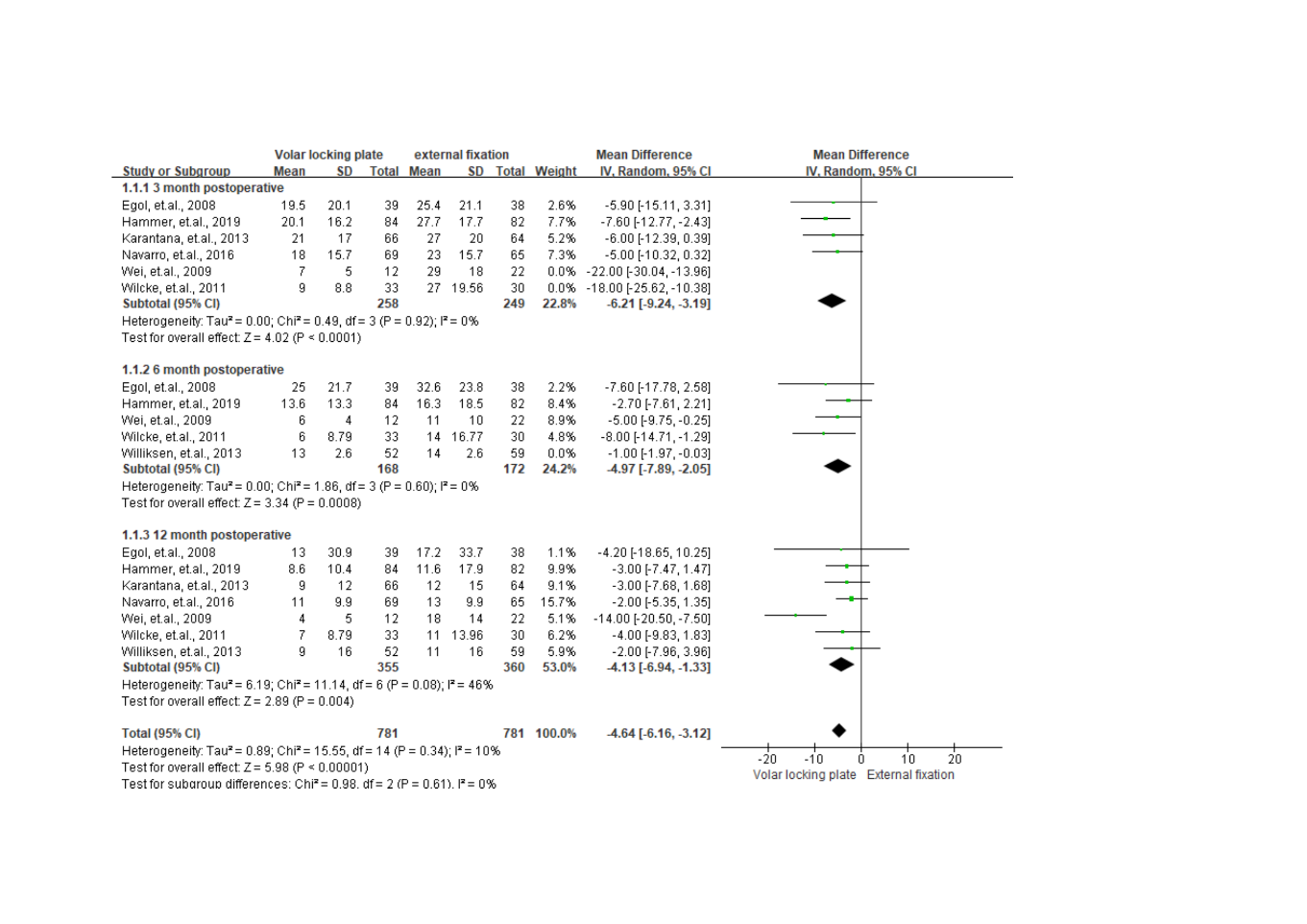


**Supplementary Figure 1**. Heterogeneity analysis for summarized disabilities of the arm shoulder and hand score


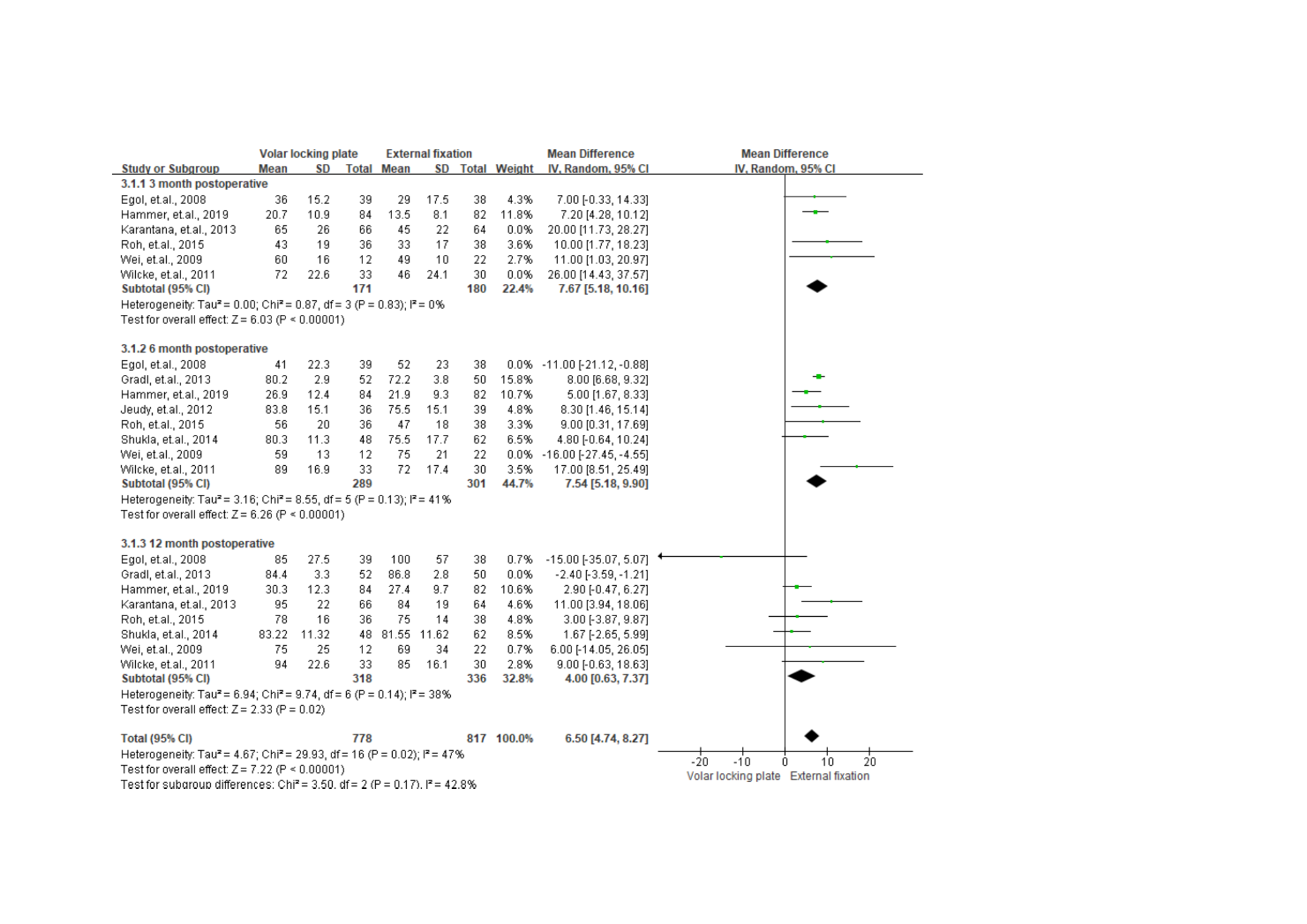


**Supplementary Figure 2.** Heterogeneity analysis for summarized grip strength


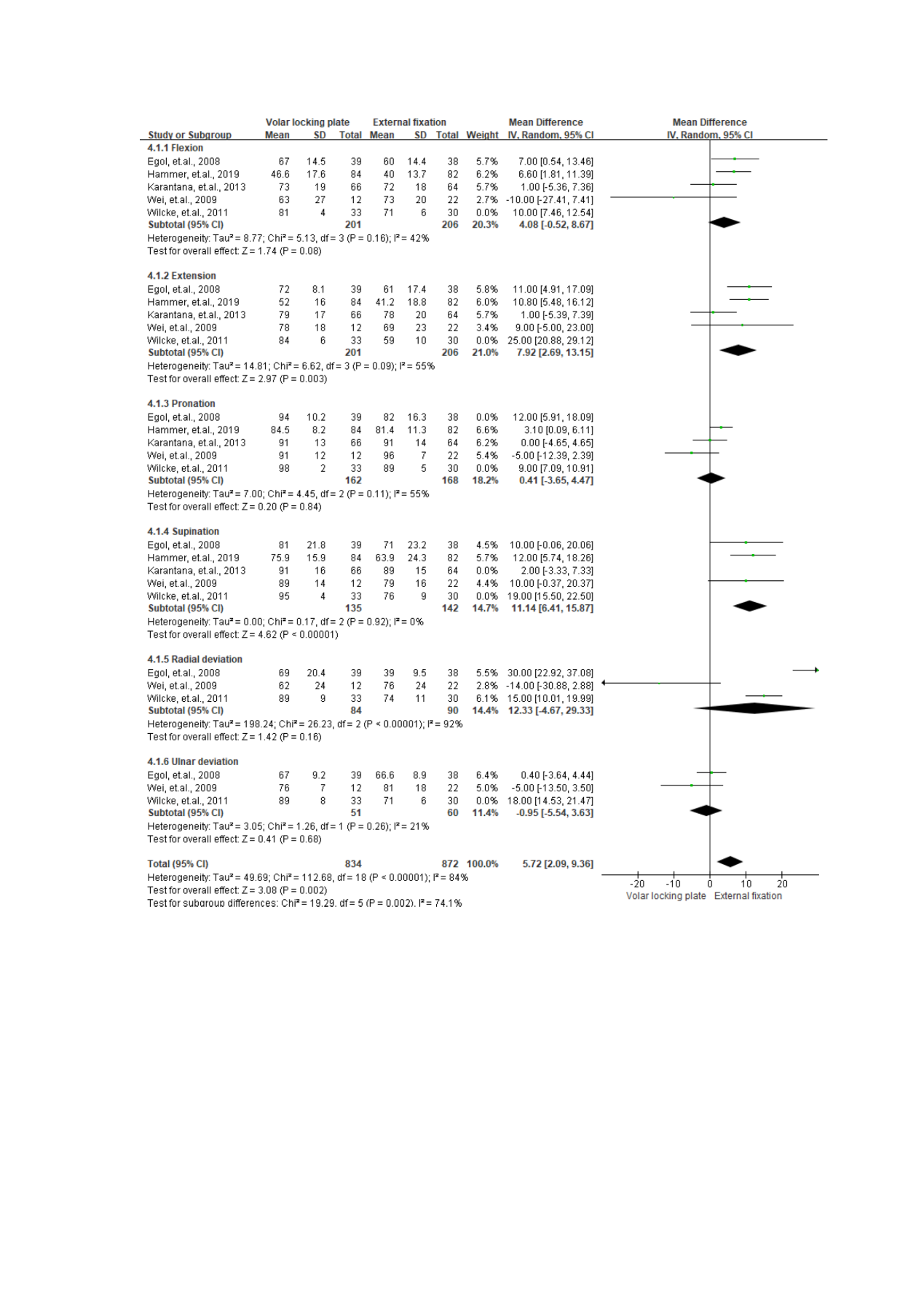


**Supplementary Figure 3.** Heterogeneity analysis for summarized wrist range of motion after 3 months


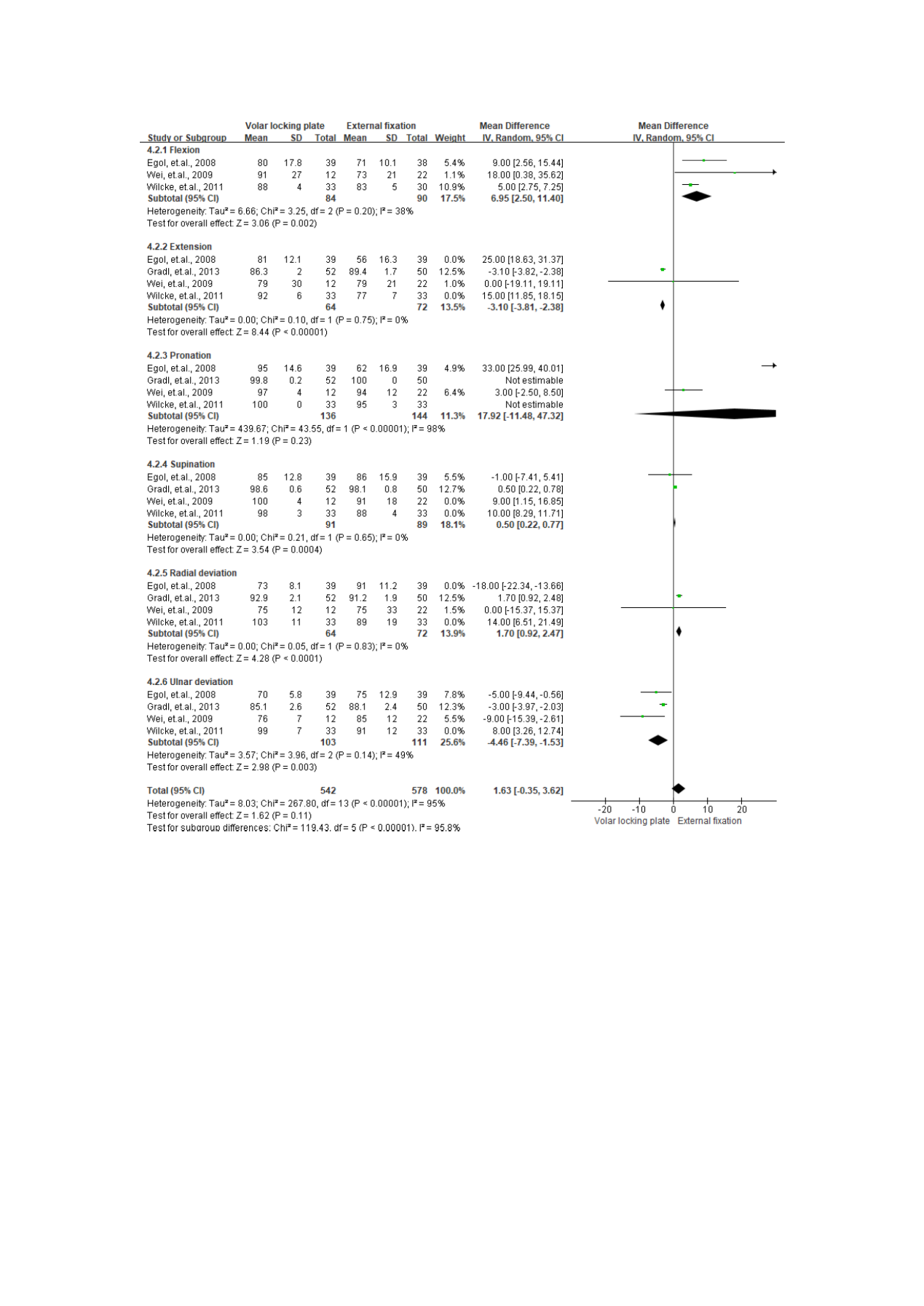


**Supplementary Figure 4.** Heterogeneity analysis for summarized wrist range of motion after 6 months


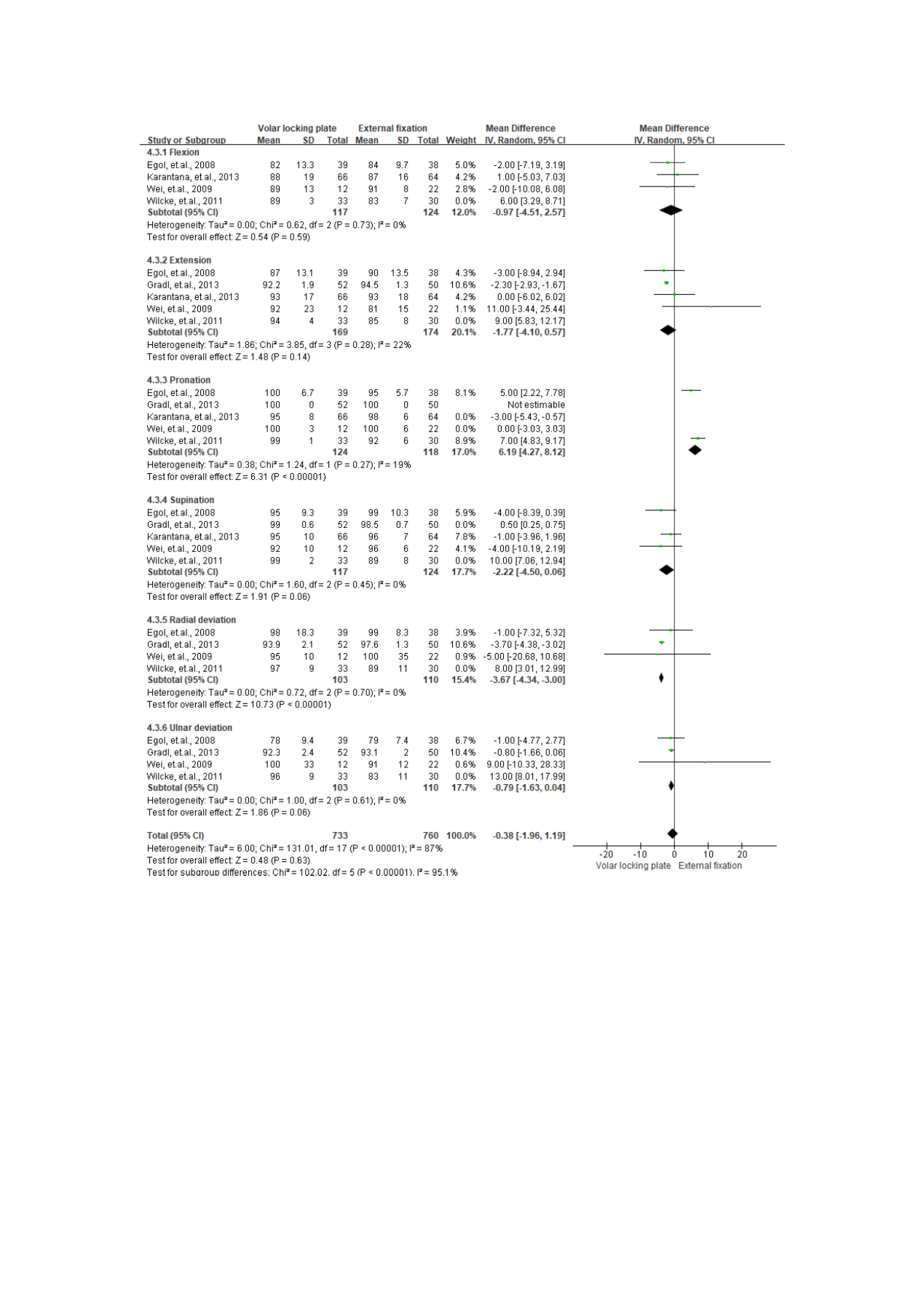


**Supplementary Figure 5.** Heterogeneity analysis for summarized wrist range of motion after 12 months


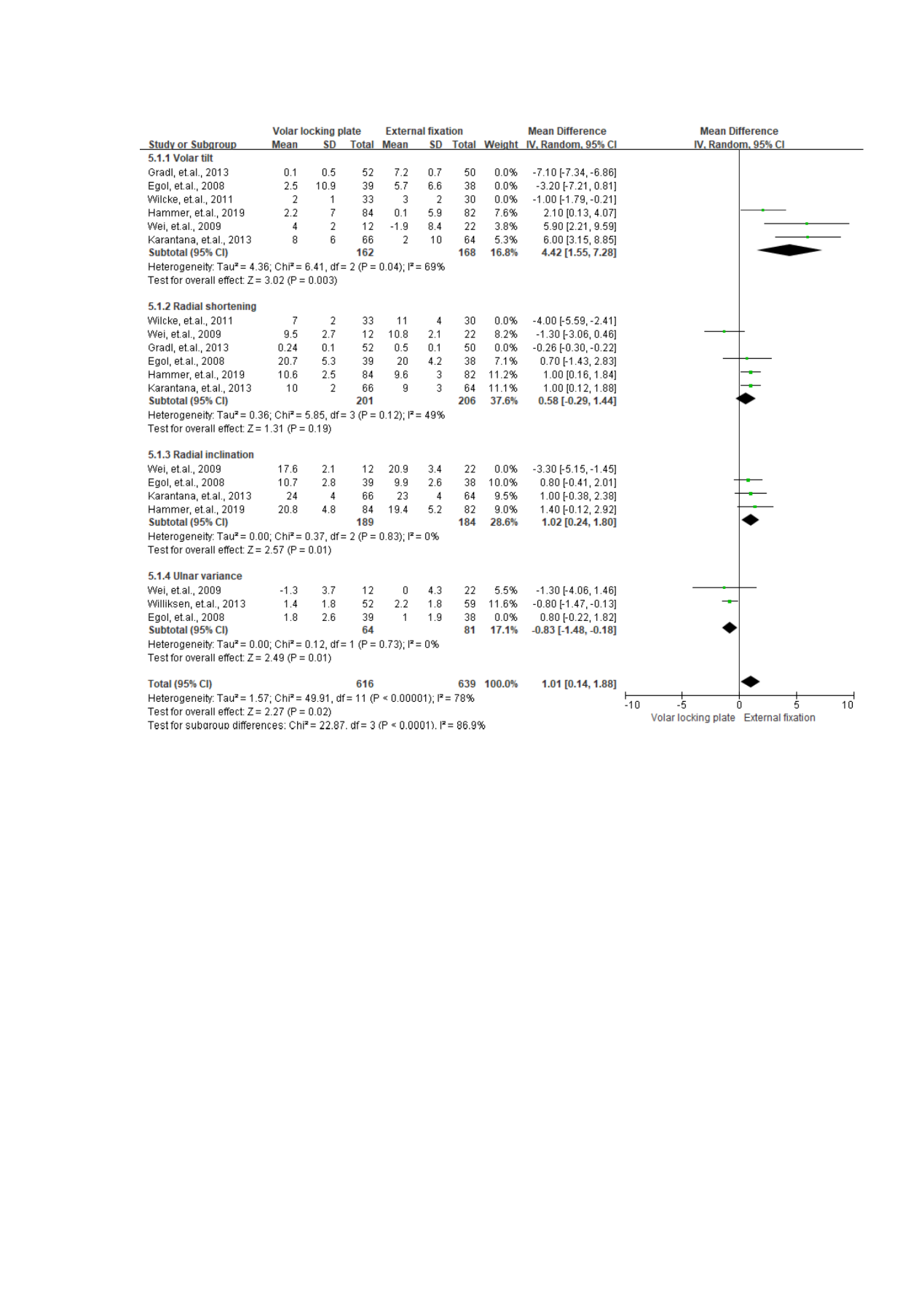


**Supplementary Figure 6.** Heterogeneity analysis for summarized radiological measurement.

**Supplementary Table 1. Quality assessment of included studies by Jadad score**

| **Study included** | **Overall quality score** |
| --- | --- |
| Egol, et.al., 2008 | 4 |
| Wei, et.al., 2009 | 4 |
| Wilcke, et.al., 2011 | 3 |
| Jeudy, et.al., 2012 | 3 |
| Gradl, et.al., 2013 | 3 |
| Karantana, et.al., 2013 | 3 |
| Williksen, et.al., 2013 | 2 |
| Shukla, et.al., 2014 | 2 |
| Roh, et.al., 2015 | 4 |
| Navarro, et.al., 2016 | 3 |
| Chung, et.al., 2019 | 3 |
| Hammer, et.al., 2019 | 4 |

**Supplementary Table 2. Publication bias of summarized outcomes**

| **Outcomes** | **Begg (*P* value)** | **Egger (*P* value)** |
| --- | --- | --- |
| Summarized disabilities of the arm shoulder and hand score after 3 months | 0.21 | 0.15 |
| Summarized disabilities of the arm shoulder and hand score after 6 months | 0.11 | 0.08 |
| Summarized disabilities of the arm shoulder and hand score after 12 months | 0.26 | 0.17 |
| Summarized visual analogue scale scores after 3 months | 0.41 | 0.33 |
| Summarized visual analogue scale scores after 6 months | 0.29 | 0.21 |
| Summarized visual analogue scale scores after 12 months | 0.19 | 0.11 |
| Summarized grip strength after 3 months | 0.66 | 0.45 |
| Summarized grip strength after 6 months | 0.48 | 0.41 |
| Summarized grip strength after 12 months | 0.32 | 0.20 |
| Summarized flexion after 3 months | 0.21 | 0.19 |
| Summarized flexion after 6 months | 0.74 | 0.69 |
| Summarized flexion after 12 months | 0.75 | 0.66 |
| Summarized extension after 3 months | 0.80 | 0.82 |
| Summarized extension after 6 months | 0.41 | 0.33 |
| Summarized extension after 12 months | 0.19 | 0.14 |
| Summarized pronation after 3 months | 0.44 | 0.31 |
| Summarized pronation after 6 months | NA | NA |
| Summarized pronation after 12 months | 0.78 | 0.75 |
| Summarized supination after 3 months | 0.80 | 0.71 |
| Summarized supination after 6 months | 0.22 | 0.14 |
| Summarized supination after 12 months | 0.67 | 0.53 |
| Summarized radial deviation after 3 months | 0.44 | 0.41 |
| Summarized radial deviation after 6 months | 0.40 | 0.47 |
| Summarized radial deviation after 12 months | 0.67 | 0.64 |
| Summarized ulnar deviation after 3 months | 0.80 | 0.54 |
| Summarized ulnar deviation after 6 months | 0.70 | 0.87 |
| Summarized ulnar deviation after 12 months | 0.81 | 0.91 |
| Summarized volar tilt after 12 months | 0.87 | 0.70 |
| Summarized radial shortening after 12 months | 0.49 | 0.31 |
| Summarized radial inclination after 12 months | 0.37 | 0.14 |
| Summarized ulnar variance after 12 months | 0.54 | 0.41 |
| Summarized minor complications | 0.94 | 0.81 |
| Summarized overall complications | 0.71 | 0.41 |
